# Emerging CNS involvement in FAP-TTR long survival patients

**DOI:** 10.1186/1750-1172-10-S1-I14

**Published:** 2015-11-02

**Authors:** Luisa Maia

**Affiliations:** 1 Neurology Department, Hospital Santo António – CHP, 4099-001 Porto, Portugal

## Background

Transthyretin-related amyloidosis (ATTR) are the most common hereditary forms of amyloidosis and Familial Amyloid Polyneuropathy (FAP) with the TTR V30M mutation, also designated as ATTR-V30M, is the most frequent form. Up until recently, despite TTR deposition in the CNS of FAP patients at a meningeal-vascular location, clinically relevant presentations were rarely recognized.

## Methods

We evaluated a series of consecutive liver transplanted patients (ATTR-V30M and non-ATTR) for the presence and type of focal neurological episodes due to CNS dysfunction (FNEs). We characterized brain neuroimaging (CT scan) in patients presenting FNEs and transthyretin amyloid deposition in the brains of ATTR-V30M autopsied patients.

## Results

We found that CNS clinical involvement occurs in FAP patients that underwent liver transplantation (LT). Patients exhibited positive and negative FNEs clinically similar to the recently described “amyloid spells” characteristic of Aβ related cerebral amyloid angiopathy (CAA) patients. Longer disease duration, male gender and renal dysfunction were associated with the presence of FNEs in FAP patients after LT. In post-mortem brain analysis, FAP patients exhibit prominent CAA associated to progressive TTR meningeal-vascular deposition, that progressed from the meninges and its vessels towards meningo-cortical vessels and the superficial brain parenchyma, as disease duration increased. Neuroimaging findings (lobar brain hemorrhage, localized subarachnoid hemorrhage and white matter damage) also support that TTR related CAA contributes to this new clinical phenotype.

## Conclusions

Both the clinic presentation and the topography of the neuropathological abnormalities of FAP patients with this new CNS phenotype overlaps to what is known to occur in sporadic Aβ-associated CAA. Moreover, neuroimaging findings in such patients suggest that CAA may be a leading player, hinting that these two pathologies could also share devastating consequences (brain hemorrhage, localized subarachnoid hemorrhage and, eventually, cognitive decline). Given the hemorrhagic risk of these vasculopathies, our results have implications in the treatment and clinical follow-up of FAP patients. In addition, sensitive imaging methods (e.g. MRI) need to be implemented in the study of ATTR patients presenting FNEs. Present and future disease modifying therapies should consider CNS TTR deposition as a target.

